# Doxorubicin-Induced Cognitive Impairment: The Mechanistic Insights

**DOI:** 10.3389/fonc.2021.673340

**Published:** 2021-05-13

**Authors:** Jiajia Du, Aoxue Zhang, Jing Li, Xin Liu, Shuai Wu, Bin Wang, Yanhong Wang, Hongyan Jia

**Affiliations:** ^1^ Department of First Clinical Medicine, Shanxi Medical University, Taiyuan, China; ^2^ Department of Breast Surgery, First Hospital of Shanxi Medical University, Taiyuan, China; ^3^ Department of Microbiology and Immunology, Shanxi Medical University, Taiyuan, China

**Keywords:** doxorubicin, cognition, mechanism, oxidative stress, inflammatory response

## Abstract

Chemotherapy can significantly prolong the survival of patients with breast cancer; Nevertheless, the majority of patients receiving chemotherapy such as doxorubicin may have cognitive deficits that manifest as impairments in learning, reasoning, attention, and memory. The phenomenon of chemotherapy-induced cognitive decline is termed as chemotherapy-related cognitive impairment (CRCI) or chemo-brain. Doxorubicin (DOX), a commonly used drug in adjuvant chemotherapy for patients with breast cancer, has been reported to induce chemo-brain through a variety of mechanisms including DNA damage, oxidative stress, inflammation, dysregulation of apoptosis and autophagy, changes in neurotransmitter levels, mitochondrial dysfunction, glial cell interactions, neurogenesis inhibition, and epigenetic factors. These mechanisms do not operate independently but are inter-related, coordinately contributing to the development of chemo-brain. Here we review the relationships of these mechanisms and pathways in attempt to provide mechanistic insights into the doxorubicin-induced cognitive impairment.

Chemotherapy is one of the effective conventional and widely used treatments for patients with cancer. Unfortunately, up to 70% of cancer patients receiving chemotherapy may develop cognitive impairment during or after treatment, which negatively affects their life-quality (1, 2). Since 1990s, it has been known that chemotherapy has adverse effects on brain function, causing dysfunctions in learning, memory, attention, motor activity, and executive function (3, 4). Numerous studies have shown that tyrosine kinases, antimetabolites, microtubule inhibitors, and alkylating agents all can induce neurotoxicity (5–7). Doxorubicin belongs to the anthracycline class and is commonly used in the adjuvant chemotherapy regimens for breast cancer (8). Doxorubicin exerts its antitumor effects through DNA insertion and inhibition of topoisomerase II. In addition, doxorubicin causes the production of invasive systemic reactive oxygen species (ROS) (9). Notably, despite its limited passage through the blood-brain barrier (BBB), doxorubicin can still cause severe neurotoxicity in the brain, and several clinical studies reported that patients of all ages treated with doxorubicin exhibited impaired ability in cognitive assessments (10–13).

Both direct or indirect mechanisms contribute to the doxorubicin-induced cognitive deficits, and the inter-relation among the multiple mechanisms are complex. A better understanding of those mechanisms and pathways and their coordinative operations may help devise novel therapeutic interventions to prevent or treat chemo-brain. The objective of this review is to comprehensively summarize and discuss the mechanisms involved in doxorubicin-induced cognitive impairment.

## Direct Neurotoxicity

**Figure 1:**
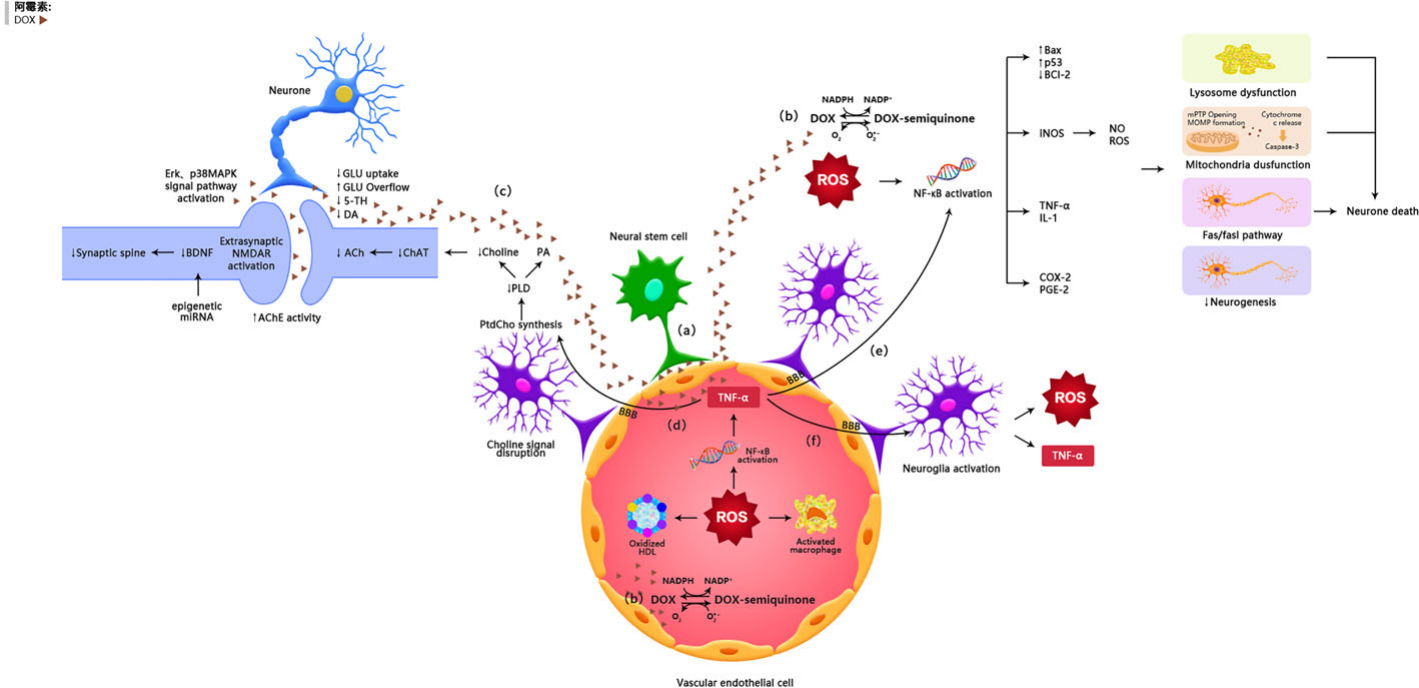
The possible mechanisms of doxorubicin-induced chemobrain. **(A)** Doxorubicin can cross the blood-brain barrier through vascular-associated apical projections of neural stem cells. **(B)** Oxidative Stress Ptahway. **(C)** Synaptic dysplasia. **(D)** TNF-α is involoved in neurotransmitter pathways. **(E)** TNF-α is involved in oxidative stress pathways. **(F)** TNF-α amplifies inflammatory signals by activating glial cells.

It was generally believed that doxorubicin has a limited capacity to penetrate the blood-brain barrier and thus the brain is protected from its damage. However, several studies have shown that doxorubicin has potential antitumor effects on brain cancer (14). Clinical as well as animal studies have shown that doxorubicin was detected in the brain after peripheral administration of the drug (15, 16). Recently, it was reported that doxorubicin could cross the blood-brain barrier through vascular-associated apical projections of neural stem cells (which are about 30 nm in diameter), can establish direct membrane-membrane contacts with the endothelial cells in specific regions of the irregular endothelial basement membrane, and have abundant vesicular activity (17). The possible direct mechanisms of doxorubicin induced chemo-brain are illustrated in **Table 1** and **Figure 1**.

**Table 1:** The direct neurotoxicity of doxorubicin on chemo-brain.

	Mechanism	Interpretation	Ref
Direct Neurotoxicity	DNA Damage and Cell Cycle Disruption	DSBs and DNA cross-linking; BRCA1 was downregulated.	Manchon JF et al. (18)
		internal or mitochondrial apoptotic pathways; caspase-dependent intrinsic apoptotic pathways.	Shokoohinia Y et al. (19)
			Lee YJ et al. (20)
			Shamas-Din A et al. (21)
		increased neuronal cell death in the early and late days.	Ramalingayya GV et al. (22)
		blocked cell cycle progression in the G2/M and S phases.	Pei Y et al. (23)
	Mitochondrial dysfunction and Increased Oxidative Stress	ROS production and mitochondrial membrane depolarization.	Shokoohinia Y et al. (19)
			Ramalingayya GV et al. (22)
		increases the Bax/Bcl-2 ratio and MOMP.	Shokoohinia Y et al. (19)
			Peng W et al. (24)
		both endogenic and ectogenic hydrogen peroxide can induce neural degeneration.	Errea O et al. (25)
		elevated mitochondrial ROS levels and calcium disorder.	Park HS et al. (26)
		glucose metabolism was decreased in both the bilateral cortex and hippocampus.	Lim I et al. (27)
		the opening of the mPTP.	Javadov S et al. (28)
			Wang CY et al. (29)
			Tangpong J et al. (30)
		interaction p53 with Bcl-xL.	Tangpong J et al. (30)
Direct Neurotoxicity	Effect on autophagic lysosomal system	impair progenitor neuronal lysosomes;promote the formation of pre-autophagic complexes.	Moruno-Manchon JF et al. (31)
	Activation of apoptosis	exogenous pathway in primary cortical neurons (death receptor-mediated” apoptosis).	Walczak H et al. (32)
	Damaged neurogenesis	reduced cell survival in the dentate gyrus and subgranular areas of rats.	Kitamura Y et al. (33)
		activation of astrocytes and subsequent release of inflammatory mediators.	Kohman RA et al. (34)
	Down-regulation of neurotransmitters	the levels of PLD, ChAT activity, and choline-containing compounds in the hippocampal region were significantly declined.	Lim I et al. (27)
			Keeney JTR et al. (35)
		doxorubicin-induced oxidative stress increased ROS-mediated AChE activity.	El-Agamy SE et al. (36)
		reduced glutamate clearance.	Thomas TC et al. (37)
		TNF-α-induced activation of astrocytes triggers substantial glutamate release.	Habbas S et al. (38)
		reduced the levels of two monoamines: 5-HT and DA.	Kwatra M et al. (39)
	Synaptic dysplasia	inhibits the growth of neurons, as evidenced by a decline in the number of neurons and a decrease in synapsin expression.	Manchon JF et al. (18)
			Ramalingayya GV et al. (22)
	Altered protein kinase signaling pathways	activate ERK and p38 MAPK.	El-Agamy SE et al. (40)
	Epigenetic alterations	miRNA dysregulation is associated with the altered levels of BDNF.	Kovalchuk A et al. (41)

DSBs, DNA double-strand breaks; BRCA1, breast cancer type 1 susceptibility protein; ROS, reactive oxygen species; MOMP, mitochondrial outer membrane permeability; mPTP, mitochondrial permeability transition pore; PLD, phospholipase D; ChAT, choline acetyltransferase; AChE, acetylcholinesterase; 5-HT, serotonin; DA, dopamine; ERK, extracellular signal-regulated kinase; BDNF, brain-derived neurotrophic factor.

### DNA Damage

An important mechanism by which doxorubicin kills cancer cells is its ability to effectively cross-link with DNA, resulting in disruption of the cell cycle and subsequent death of cancer cells (23, 42). However, doxorubicin can also damage normal and non-cancerous cells (43).

Manchon et al. proved that doxorubicin accumulated in the nucleus of neurons, leading to DNA double-strand breaks (DSBs) and DNA cross-linking. Furthermore, breast cancer type 1 susceptibility protein (BRCA1), which is responsible for DNA repair, was downregulated in primary cortical neurons after doxorubicin treatment (18). DNA fragmentation following doxorubicin treatment is a strong stimulus to incur internal or mitochondrial apoptotic pathways *via* increasing the Bax/Bcl-2 ratio; in addition, it can assemble the bax - bax oligomers on mitochondrial membranes, resulting in increased mitochondrial outer membrane permeability (MOMP) and the release of cytochrome C, and activating the caspase-dependent intrinsic apoptotic pathways (19, 44, 45). Also, it was showed that doxorubicin remarkably increased neuronal cell death in the early and late days (22). In a human neuroblastoma model, doxorubicin prevented cell cycle progression in the G2/M and S phases (23).

The death of neurons seriously affects the normal activities of the brain, which is manifested by impaired cognitive functions such as learning and memory.

### Increased Oxidative Stress

Doxorubicin causes neurotoxicity by facilitating ROS production and mitochondrial membrane depolarization in neurons (19, 22). Doxorubicin increases the MOMP and Bax/Bcl-2 ratio, leading to mitochondrial degeneration and neuronal dysfunction (19, 24). Previous studies have shown that both endogenic and ectogenic hydrogen peroxide can induce neural degeneration (25).

Except for being a site of ATP production, mitochondria are the main organelles which adjust calcium absorption and redox signaling under physiological conditions (46). It was found that doxorubicin damaged mitochondrial function in the hippocampus, resulting in elevated mitochondrial ROS levels and calcium disorder (26). In addition, glucose metabolism was declined in both the hippocampus and bilateral cortex after intra-thecal injection of doxorubicin (27). This damage may be generated by the opening of the mitochondrial permeability transition pore (mPTP), which is assembled between the mitochondrial membranes by three protein subunits including cyclophilin D (CyP-D), adenine nucleotide translocase (ANT), and VDAC (47). ROS initiates the activation of glycogen synthase kinase-3, which phosphorylates CyP-D into its active form. In addition, Calcium dysregulation contributes to mitochondrial membrane depolarization and ANT conformational changes. All these contribute to the mPTP formation, leading to abnormal solute divulgation and mitochondrial swelling (29, 47). Interestingly, injection of the antibodies against TNF-α or iNOS totally prevented the damage of mitochondrial oxidative reaction in mice, suggesting that doxorubicin reduces the mitochondrial function *via* inflammatory reaction and NO• production (30).

Tangpong et al. showed that doxorubicin shifted p53 to the outer mitochondrial membrane, promoting its interaction with Bcl-xL (30), and this interplay caused the separation of Bax from Bcl-2 and Bcl-xL, led to Bax oligomerization and increased MOMP, resulting in the release of cytochrome C (30).

Mitochondria are the energy source of neurons, carrying out countless REDOX reactions all the time. Oxidative stress and neuron degeneration caused by abnormal mitochondria are one of the important causes of cognitive impairment (22).

### Effect on Autophagic Lysosomal System

Doxorubicin can cause damage to the progenitor neuronal degradation pathways, impair progenitor neuronal lysosomes, promote the formation of pre-autophagic complexes, up-regulate autophagy, and affect the clearance of the autophagic marker p62 protein (31). Under electron microscopy, an accumulation of vacuolar structures, autophagosomes, mitochondria, and lipid droplets was observed in doxorubicin- exposed neurons (31).

Degradation disorders seriously affect the function of neurons, resulting in cognitive impairment after chemotherapy (31).

### Activation of Apoptosis

It has been noted that doxorubicin-induced apoptosis is dependent on the exogenous pathway in primary cortical neurons (death receptor-mediated” apoptosis). Exogenous apoptotic pathway is a direct apoptotic mechanism mediated by doxorubicin. Doxorubicin increases Fas-Fas ligand (FasL) interactions, leading to the recruitment of Fas-associated protein with death domain (FADD) by connecting to the death domain, which initiates exogenous apoptotic pathways (32). Endogenous apoptotic pathways are activated by cellular stress, DNA damage, developmental signaling, and loss of survival factors. This pathway is regulated by Bcl-2 family proteins, and is related to the mechanism of mitochondrial oxidative stress, which has been described in detail above.

Abnormal apoptosis greatly reduces the number of neurons, thus leading to cognitive impairment (32).

### Damaged Neurogenesis

The hippocampus is one of the structures closely associated with spatial processing and memory formation, and the integration of mature neurons in the circuit plays a key role in hippocampal neurogenesis (48). Animals treated with doxorubicin showed an obvious decrease in neurogenesis, as manifested by a distinct reduction in the number of neuro-specific nuclear antigen bromodeoxyuridine (BrdUrd)-labeled cells (49). Others also found that DOX in combination with cyclophosphamide reduced cell survival in the subgranular areas and dentate gyrus of rats (33).

A large number of studies have shown that activation of astrocytes and subsequent release of inflammatory mediators caused by doxorubicin render the nerve non-viable (50). TNF-α was reported to be anti-neurogenic, and can cause a decrease of the BrdUrd-labeled cells in the sub-granular zone following injection (51). Besides, mice deficient in TNF-α-receptor-1 (TNFR1) had an increased proliferation of BrdUrd-labeled cells in the sub-granular compartment, suggesting that TNFR1 mediates the anti-neurogenic effects of TNF-α (52). Neuro-inflammation not only affects the proliferation, differentiation, and survival of hippocampal cells, but also prevents the incorporation of new neurons into existing neural networks (53).

### Down-Regulation of Neurotransmitters

Many animal researches have manifested that doxorubicin can cause dysregulation of neurotransmitter production and release in the brain. Acetylcholine (ACH) is a significant neurotransmitter in the cholinergic nervous system that supports brain functions through long-term potentiation (LTP) (54). During acetylcholine composition, phosphatidylcholine (PtdCho) is disintegrated by phospholipase D (PLD) and this releases choline, which is acetylated by choline acetyltransferase (ChAT) to form acetylcholine (55). In mice, the levels of PLD, ChAT activity, and choline-containing compounds in the hippocampal region were significantly declined after doxorubicin treatment, reflecting the exhaustion of ACH production (27) (35). Moreover, doxorubicin-induced oxidative stress increased ROS-mediated acetylcholinesterase (AChE) activity (36). Changes in the choline-containing substances are thought to be related to membrane turnover (synthesis and degradation of phospholipids), and have been attributed to myelin injury following chemotherapy (15).

Elevated TNF-α may decrease PLD activity, thereby inhibiting PtdCho synthesis (56). In addition, TNF-α is thought to be associated with decreases of phosphatidic acid levels, suggesting an interdependence between phospholipase and TNF-α expression (57).

Inhibition of PLD leads to decreased production of cytokines, including TNF-α (58, 59). Phosphatidic acid, an intermediate product of the PLD pathway, stimulates Ca2+ mobilization and displays growth factor-like activity, which helps reduce doxorubicin-induced mitochondrial dysfunction in the mouse brain (60). The enzymatic activity of PLD is critical for cell survival, and structural damage to PLD and reduced PLD activity may activate apoptotic pathways (37, 38, 61).

In addition to regulating acetylcholine metabolism, doxorubicin can alter glutamate levels in the synaptic gap. Doxorubicin reduced glutamate clearance, as showed by a decline in the rate of uptake of glutamate in the frontal cortex of mice (62). In this context, it was suggested that the decreased glutamate clearance is due to decreased expression of glial transport proteins or increased glutamate production from neurogliocyte, particularly in astrocytes (62). As mentioned earlier, TNF-α-induced activation of astrocytes triggers substantial glutamate release (63). When glutamate concentrations are high in the synapse, glutamate can diffuse to and combine with NMDA acceptors. Activation of extrasynaptic NMDA acceptors causes increased calcium-dependent excitability and suppression of BDNF composition, leading to loss of synaptic plasticity and increased neuronal apoptosis (39, 64). This may explain how doxorubicin decreased the expression of BDNF and its receptor tropomyosin receptor kinase B (TrkB) (26).

In addition, doxorubicin injection distinctly reduced the levels of two monoamines that are closely related to cognitive function: serotonin (5-HT) and dopamine (DA) (65). 5-HTergic neurons play a significant role in regulating hippocampal synaptic plasticity through 5-HT1A receptor-mediated inhibitory control. Depletion of 5-HT negatively affects hippocampus-dependent declarative memory and performs poorly in a new object recognition task (66). During encoding, doxorubicin mediates the acquisition of long-lasting, long-term memory in the hippocampus by activating the D1/D5 receptor (67).

Changes in the levels of neurotransmitters lead to abnormalities in the transduction of nerve signals, leading to cognitive impairment.

### Synaptic Dysplasia

Abnormal synaptic plasticity in the brain is an important cause of cognitive impairment. Synaptic plasticity is associated with synapse-associated proteins such as synapsin protein (SYP) and postsynaptic dense protein 95 (PSD95). In addition, the expression of brain-derived neurotrophic factor (BDNF)-synuclein (SYP)-microtubule-associated protein 2 (MAP2) pathway-related proteins in the hippocampus is also involved in the development of synaptic plasticity. Doxorubicin not only causes chromatin condensation and cell membrane fragmentation, but also inhibits the growth of neurons, as evidenced by a decline in the number of neurons and a decrease in synapsin expression, resulting in cognitive impairment (18, 22).

### Altered Protein Kinase Signaling Pathways

Doxorubicin can affect some key memory-related kinase systems. For instance, doxorubicin can activate p38 MAPK and extracellular signal-regulated kinase (ERK), two kinases that have opposite roles: while the former mediates synaptic inhibition, the latter promotes synaptic facilitation (40).

In hippocampal sensory neurons, doxorubicin can inhibit serotonin-induced long-term facilitation (LTF) and promote Phe-Met-Arg-Phe-NH2 (FMRFa)-mediated long-term depression (LTD), suggesting that doxorubicin may block learning-related changes in hippocampal excitability (40). However, p38-mediated inhibition of LTF was superior to ERK effects (40). The deviation of LTF was corrected by p38 inhibitors (68). These studies mean that long-term memory damage may be the result of doxorubicin action, partially due to the dominant activation of p38 MAPK (68). In addition, doxorubicin inhibited the phosphorylation of the downstream transcriptional repressor cAMP response element binding protein 2 (CREB2), which promoted LTD (40).

The ERK pathway is essential for neuronal survival (69) and is required for the synthesis of Arc, a protein that plays a crucial part in long-term memory formation, neuronal activity and synaptic plasticity (41). A recent study found an increase in Arc staining after doxorubicin treatment, suggesting that doxorubicin induces neuronal activity. On the contrary, inhibition of neural activity with n -methyl-d-aspartate (NMDA) receptor antagonists or α-amino-3-hydroxy-5-methyl-4-isoxazolepropionic acid (AMPA) receptor antagonists partly eliminated the induction of DNA DSBs by doxorubicin, suggesting that doxorubicin-induced neurotoxicity is dependent on neuronal activity (18).

### Epigenetic Alterations

It has been reported that chemotherapy can trigger epigenetic reprogramming, another important mechanism that may contribute to persistent cognitive impairment (70). Homozygous mice exposed to chemotherapeutic agents show more pronounced disruptions in post-transcriptional regulation of gene expression, mainly miRNA changes in the prefrontal cortices. miRNA dysregulation is associated with the altered levels of brain-derived neurotrophic factor (BDNF) that plays a key role in cognition and memory (71).

## Indirect Neurotoxicity

Doxorubicin itself has a limited capacity to penetrate the blood-brain barrier; however, in the periphery, this agent can induce the production of a number of inflammatory factors and neurotransmitters that can pass through the BBB, thus affecting neurogenesis and survival, suggesting indirect neurotoxicity. The possible indirect mechanisms of doxorubicin induced chemo-brain are illustrated in **Table 2** and **Figure 1**.

**Table 2:** The indirect neurotoxicity of doxorubicin on chemo-brain.

	Mechanism	Interpretation	Ref
Indirect Neurotoxicity	Induction of oxidative Stress	Excess ROS production leads to the oxidative modification of biochemical molecules such as proteins, lipids, and nucleic acids.	Birben E et al. (72)
		ROS can activate NF-κB.	Herb M et al. (73)
			Yan S et al. (74)
	Inflammation	TNF-α can affect the volume of the hippocampus.	Kwatra M et al. (39)
		TNF-α can inhibit the long-term enhancing effects of hippocampal CA1 and the dentate gyrus.	Motaghinejad M et al. (75)
		TNF-α can augment the inflammatory signals by activating astrocytes and microglia, which lead to the local production of TNF-α in the brain.	Guidotti G et al. (76)
			Zhou H et al. (77)
		Binding of TNF-α to TNFR recruits intracellular proteins and transduces inflammatory signaling, leading to NF-κB translocates to the nucleus.	Mohamed RH et al. (78)
			Wu YQ et al. (79)
		activation of microglia and astrocytes *via* TNFR1 can enhance the expression and activity of NOX, particularly NOX2, leading to an increase in ROS production.	Blaser H et al. (80)
Indirect Neurotoxicity	Nitrification Stress	the nitrated MnSOD resulted in impaired mitochondrial respiratory activity, which in turn synergized with O2•− production.	Tangpong J et al. (30)
			Holley et al. (81)
	Apolipoprotein A-I	ApoA-1 exerts anti-inflammatory effects by blocking contact between activated T lymphocytes and monocytes and inhibiting the production of TNF-α.	Ronkina N et al. (82)
		ApoA-1 is sensitive to the doxorubicin-induced oxidative damage, leading to dyslipidemia and increased circulating TNF-α.	Ramalingayya GV et al. (22)
			Aluise CD et al. (70)
			Tangpong J et al. (83)

NF-κB, nuclear factor-κB; TNFR, TNF-α receptor; TNFR1, TNF-α receptor 1; NOX, NADPH oxidases; MnSOD:manganese superoxide dismutase; ApoA-1, Apolipoprotein A-I.

### Induction of Oxidative Stress

Peripheral inflammation and oxidative stress are believed to be responsible, at least in part, for doxorubicin-induced chemo-brain pathogenesis (30, 35, 84). Doxorubicin is a quinone-containing compound, a structure that is readily reduced by a single electron, and can be converted to semiquinone radicals by NADPH cytochrome P450 reductase (85), NADH dehydrogenase (mitochondrial complex I) (86), and cytoplasmic xanthine oxidase (87). The semiquinone form of doxorubicin can react with oxygen molecules and return to the natural quinone form, while forming a superoxide anion radical (O2•−). This process is repeated following doxorubicin injection and is known as the redox cycle (72). O2•− is a secondary product of ROS, including hydrogen peroxide (H2O2) and hydroxyl radicals (•OH), which can cause peripheral oxidative stress (73). Excess ROS production leads to the oxidative modification of biochemical molecules such as proteins, nucleic acids, and lipids (74). In clinical researches, doxorubicin-mediated increases of oxidative stress were indicated by increased protein oxidation and lipid peroxidation as well as decreased levels of enzymatic and nonenzymatic antioxidants (35, 84).

ROS can activate nuclear factor-κB (NF-κB), a redox-sensitive transcription factor, through a classical IκB kinase (IKK)-dependent pathway (88, 89). NF-κB activation enhances the expression of several pro-inflammatory cytokines, including tumor necrosis factor-α (TNF-α) (83), interleukin-1β (IL-1β), and interleukin-6 (IL-6) (90). It has been demonstrated that doxorubicin injection into mice caused an increase in plasma TNF-α levels 1 hour after the treatment (75). Similarly, serum cultures of isolated macrophages from the doxorubicin-treated mice show elevated levels of TNF-α (91). This suggests an interaction between oxidative stress and inflammatory factors.

Neuronal degeneration caused by oxidative stress is one of the important causes of cognitive impairment (91).

### Inflammation

TNF-α can affect the volume of the hippocampus (65) and inhibit the long-term enhancing effects of hippocampal CA1 and the dentate gyrus (92). Circulating TNF-α is able to migrate into the brain *via* endothelial cells expressing TNF-α receptors 1 and 2 (TNFR1 and TNFR2) in the BBB (76, 77). Once in the brain and upon binding to receptors on glial cells, TNF-α can augment the inflammatory signals by activating astrocytes and microglia, which lead to the local production of TNF-α in the brain (78, 79). Binding of TNF-α to TNFR recruits intracellular proteins and transduces inflammatory signaling, leading to NF-κB translocating to the nucleus (80, 93).

In addition to inflammatory cytokines, activation of microglia and astrocytes *via* TNF-α receptor 1 (TNFR1) can enhance the expression and activity of NADPH oxidases (NOX), particularly NOX2, leading to an increase in ROS production (94). Moreover, brain inflammation can down-regulate the expression of inducible nitric oxide synthase (iNOS) through NF-κB transcriptional activity, causing oxidative and nitrification stress in the brain (95, 96).

Inflammatory factors and oxides interact with each other to strengthen each other, affecting the shape and function of the hippocampus, causing cognitive impairment (93).

### Nitrification Stress

Several *in vivo* studies have shown that doxorubicin can induce iNOS expression and elevate levels of nitric oxide (NO•) in the body (80, 91, 93). It has been demonstrated that high concentrations of NO• can react with O2•− to form peroxynitrite (ONOO), one of the most detrimental oxidants (81). In the mice treated with doxorubicin, both NO• and ONOO− cause nitrification by adding a 3-nitrotyrosine (3-NT) to the protein (91) (97). Studies have shown that an antioxidant enzyme, manganese superoxide dismutase (MnSOD), was nitrated in the mitochondria in the animals subjected to doxorubicin treatment, and the nitrated MnSOD resulted in impaired mitochondrial respiratory activity, which in turn synergized with O2•− production (30, 82). Inflammatory processes and oxidative stress may exacerbate doxorubicin-induced neurotoxicity, and ultimately lead to neuronal apoptosis in the neurogenic regions of the brain.

### Apolipoprotein A-I

Apolipoprotein A-I (ApoA-1) is the primary lipoprotein component of high-density lipoprotein cholesterol (HDLeC) and has important biological functions (98).

Besides removing excess cholesterol through ATP-binding cassette protein 1 (ABCA1), ApoA-1 exerts anti-inflammatory effects by blocking contact between activated T lymphocytes and monocytes and inhibiting the production of TNF-α, which is achieved through up-regulating the production of an mRNA-unstable protein, tristetraprolin. Tristetraprolin can induce the disassembly of TNF-deletion factor mRNA, thereby inhibiting TNF-deletion factor translocation (99). Similar to other biochemicals in the circulation, ApoA-1 is sensitive to the doxorubicin-induced oxidative damage (84), leading to dyslipidemia and increased circulating TNF-α in the doxorubicin- injection animals (22, 75), resulting in cognitive impairment (84).

## Conclusions and Prospect

It has been confirmed by a number of clinical as well as pre-clinical investigations that chemotherapy may cause cognitive impairment, and the mechanisms involved in the chemo-brain problem include DNA damage, oxidative stress, inflammatory responses, dysregulation of apoptosis and autophagy, altered neurotransmitter levels, aberration of some key kinases, mitochondrial dysfunction, glial cell interactions, inhibition of neurogenesis, and epigenetic factors. Noteworthily, all of these mechanisms are inter-related and collectively involved in the development of the chemo-brain. Although there is no specific treatment for chemotherapy-induced cognitive impairment, it has been reported that rehabilitation behavioral training, such as cognitive behavioral therapy (CBT), neuropsychological/cognitive training intervention, physical activity, might improve the quality of life of the patients who suffer from chemo-brain. In addition, because doxorubicin has limited ability to penetrate through the BBB and key factors in the induction of chemo-brain are oxidative stress and peripheral TNF-α products, antioxidant or anti-inflammatory therapy often can significantly improves chemo-brain. In fact, several pharmacological agents have shown promising benefits in blocking neurotoxic pathways through their antioxidant or anti-inflammatory action, such as 2-mercaptoethane sulfonate sodium(MESNA) (100), donepezil (27), astaxanthin(AST) (36); however, their impact on the antitumor efficacy of chemotherapy regimens remains to be evaluated.

Chemotherapy is one of the most important modalities in the comprehensive treatment of breast cancer. In order to improve the therapeutic effect, chemotherapy regimens combining two or more drugs or sequential chemotherapy are often used in clinical practice. This review focuses on the mechanism of action of doxorubicin in causing cognitive impairment, yet, there are numerous studies showing that paclitaxel, cyclophosphamide, and platinum drugs are also associated with chemotherapy brain. We speculate that the cognitive deficits caused by these chemotherapeutic drugs also have multiple cross-linked mechanisms, such as pterostilbene causes neuronal abnormalities, apoptosis, neuroinflammation, and endoplasmic reticulum stress; cyclophosphamide induces dendritic abnormalities, oxidative damage, and DNA methylation in hippocampal granule cells; and platinum drugs affect brain glucose metabolism and cause mitochondrial damage. When different drugs are used in combination or sequentially, the mechanisms leading to chemotherapy of the brain become more complex. Therefore, more animal experiments and clinical studies are needed to elucidate the network of these mechanisms related to chemo-brain, to provide a theoretical basis for the treatment and prevention of cognitive disorders caused by different chemotherapy regimens, and to hopefully achieve evidence-based, precision medicine for chemotherapy-related cognitive disorders in the near future.

During our research, we found that cognitive function is highly susceptible to subjective factors, and that stress and negative emotions affect the brain by disrupting the body’s hormonal homeostasis. In addition, cancer, general anesthesia, and surgery can increase cytokine levels and cause cognitive impairment. Therefore, in future research, we should pay special attention to the selection of subjects and the design of experimental protocols. Currently, there is no unified standard for the diagnosis of chemotherapy-related cognitive impairment at home and abroad. Examination methods mainly include neuropsychological testing and imaging examinations. The Functional Assessment of Cancer Therapy–Cognitive Function (FACT-Cog) is a scale developed by Wagner et al. for the evaluation of subjective cognitive impairment in cancer patients, which has been proved to have good reliability and validity for breast cancer patients in China. Montreal Cognitive Assessment (MoCA) is an assessment tool for rapid screening of objective cognitive dysfunction, which is suitable for clinical application due to its high sensitivity and short test time for mild cognitive impairment. However, these neuropsychological tests are highly susceptible to subjective factors such as stress and negative emotions, and need to refer to objective cognitive assessment methods such as MRI. In recent years, multimodal magnetic resonance imaging (MRI) such as arterial spin labeling (ASL), blood oxygen level dependent (BOLD) functional MRI (fMRI), and diffusion tensor imaging (DTI) have been widely used to evaluate chemotherapy-induced breast cancer. This set of techniques significantly improves our understanding of the neural mechanisms underlying chemotherapy-induced cognitive dysfunction from a holistic and local brain structure and function perspective.

## Author Contributions

JD reviewed the mechanisms of doxorubicin-induced cognitive impairment, and was a major contributor in writing the manuscript. AZ revised the manuscript. JL analyzed and interpreted the patient data regarding the chemotherapy. HJ helped perform the analysis with constructive discussions. XL, SW, BW, and YW helped perform the analysis with constructive discussions. All authors contributed to the article and approved the submitted version.

## Funding

The research fund was provided by General Project of Natural Science Foundation of Shanxi Province (201901D111347).

## Conflict of Interest

The authors declare that the research was conducted in the absence of any commercial or financial relationships that could be construed as a potential conflict of interest.

## Abbreviations

DSBs, DNA double-strand breaks; BRCA1, breast cancer type 1 susceptibility protein; ROS, reactive oxygen species; MOMP, mitochondrial outer membrane permeability; mPTP, mitochondrial permeability transition pore; PLD, phospholipase D; ChAT, choline acetyltransferase; AChE, acetylcholinesterase; 5-HT, serotonin; DA, dopamine; ERK, extracellular signal-regulated kinase; BDNF, brain-derived neurotrophic factor; NF-κB, nuclear factor-κB; TNFR, TNF-α receptor; TNFR1, TNF-α receptor 1; TNFR2, TNF-α receptor 2; NOX, NADPH oxidases; MnSOD, manganese superoxide dismutase; ApoA-1, Apolipoprotein A-I.
